# The adaptation of *Escherichia coli* cells grown in simulated microgravity for an extended period is both phenotypic and genomic

**DOI:** 10.1038/s41526-017-0020-1

**Published:** 2017-05-23

**Authors:** Madhan R. Tirumalai, Fathi Karouia, Quyen Tran, Victor G. Stepanov, Rebekah J. Bruce, C. Mark Ott, Duane L. Pierson, George E. Fox

**Affiliations:** 1 Department of Biology & Biochemistry, University of Houston, Houston, TX USA; 2 NASA Ames Research Center, Moffett Field, CA USA; 3 NASA Lyndon B. Johnson Space Center, Houston, TX USA

## Abstract

Microorganisms impact spaceflight in a variety of ways. They play a positive role in biological systems, such as waste water treatment but can be problematic through buildups of biofilms that can affect advanced life support. Of special concern is the possibility that during extended missions, the microgravity environment will provide positive selection for undesirable genomic changes. Such changes could affect microbial antibiotic sensitivity and possibly pathogenicity. To evaluate this possibility, *Escherichia coli* (lac plus) cells were grown for over 1000 generations on Luria Broth medium under low-shear modeled microgravity conditions in a high aspect rotating vessel. This is the first study of its kind to grow bacteria for multiple generations over an extended period under low-shear modeled microgravity. Comparisons were made to a non-adaptive control strain using growth competitions. After 1000 generations, the final low-shear modeled microgravity-adapted strain readily outcompeted the unadapted lac minus strain. A portion of this advantage was maintained when the low-shear modeled microgravity strain was first grown in a shake flask environment for 10, 20, or 30 generations of growth. Genomic sequencing of the 1000 generation strain revealed 16 mutations. Of the five changes affecting codons, none were neutral. It is not clear how significant these mutations are as individual changes or as a group. It is concluded that part of the long-term adaptation to low-shear modeled microgravity is likely genomic. The strain was monitored for acquisition of antibiotic resistance by VITEK analysis throughout the adaptation period. Despite the evidence of genomic adaptation, resistance to a variety of antibiotics was never observed.

## Evolution: Bacteria gain advantageous mutations under simulated microgravity

Bacteria grown for an extended period of time under simulated microgravity adopt growth advantages. George Fox and colleagues from the University of Houston, Texas, USA, cultured *Escherichia coli* bacteria for 1000 generations in a high aspect rotating vessel to simulate the low fluid shear microgravity environment encountered during spaceflight. They then performed growth competition assays and found that the 1000-generation adapted bacteria outcompeted control bacteria grown without simulated microgravity. Genomic sequencing of the adapted bacteria revealed 16 mutations, five of which altered protein sequences. These DNA changes likely explain the growth advantage of the bacteria grown for multiple generations in simulated microgravity. Similar adaptations during prolonged space missions could result in nastier pathogens that might threaten the health of astronauts. Fortunately, the microbes did not appear to acquire antibiotic resistance over the 1000 generation in the modeled microgravity culture.

## Introduction

Human space travelers experience a unique environment that affects homeostasis and physiologic adaptation. Space flight has been shown to induce varied immune responses, many of them potentially detrimental.^1^ Impairment of the immune system may lead to higher risk of bacterial and/or viral infection during human space flight missions.^2–4^ In combination with this increased human risk, bacteria exposed to the same environments may become more virulent and/or antibiotic resistant as a result of exposure to the low shear environment induced.^5–7^

To better understand the effect of the space environment on microorganisms, a number of spaceflight^8–17^ and laboratory studies utilizing a low-shear modeled microgravity (LSMMG) environment have been undertaken.^5–7, 13, 18–36^ These experiments have shown changes in phenotypic microbial characteristics including microbial growth, morphology, metabolism, genetic transfer, stress susceptibility, and/or virulence in a variety of organisms including *Salmonella enterica* serovar typhimurium,^8, 9, 31, 33, 35^ *Yersinia pestis*,^5, 10, 30^ *Escherichia coli*,^19, 20, 22, 29, 34, 36^ *Pseudomonas aeruginosa*,^12, 16, 17^ *Streptococcus mutans*,^37^ *Mycobacterium marinum* ^38^ and *Candida albicans*.^11, 18, 32^ In an earlier study with *Escherichia coli (E. coli)*, we carried out transcriptional studies on the K12 MG1655 strain grown in both rich and minimal medium under LSMMG.^34^ The results were compared to a normal gravity control. This study revealed reproducible changes in transcription. However, no specific LSMMG responsive genes were identified. Instead, reduction of fluid shear and a randomized gravity vector appeared to cause local extra-cellular environmental changes, which elicit cellular responses. Thus, even in the absence of significant genomic adaptation, an organism may be more virulent simply because it is already expressing genes that facilitate avoidance of the immune system^17^ or because genes associated with virulence are being expressed.^21^

LSMMG studies to date have been short-term, thereby providing only limited opportunity for genomic changes that might make observed responses permanent. The objective of the current study was to examine the extent to which extended exposure to the LSMMG environment leads to genomic adaptation. To address this, *E. coli* MG1655 cells were exposed to simulated microgravity conditions for 1000 generations (1000G) in rich medium. The subsequent analysis used competition assays and genomic sequencing to assess the adaptation of the 1000G strains. In addition, resistance to antibiotics was monitored throughout the adaptation period.

## Results

### Competition assays

In order to assess whether genomic changes account for a portion of the adaptation, erasure studies were conducted. The 1000G-adapted strain was grown in shake flasks under normal gravity for various periods of time. If the adaptation to the high aspect rotating vessel (HARV) environment, which was used to create the LSMMG conditions, was primarily phenotypic, the favorable changes acquired during HARV growth would be rapidly erased upon return to the shake flask environment. In contrast, genomic adaptations to the HARV environment rather than being erased would persist.

**Fig. 1:**
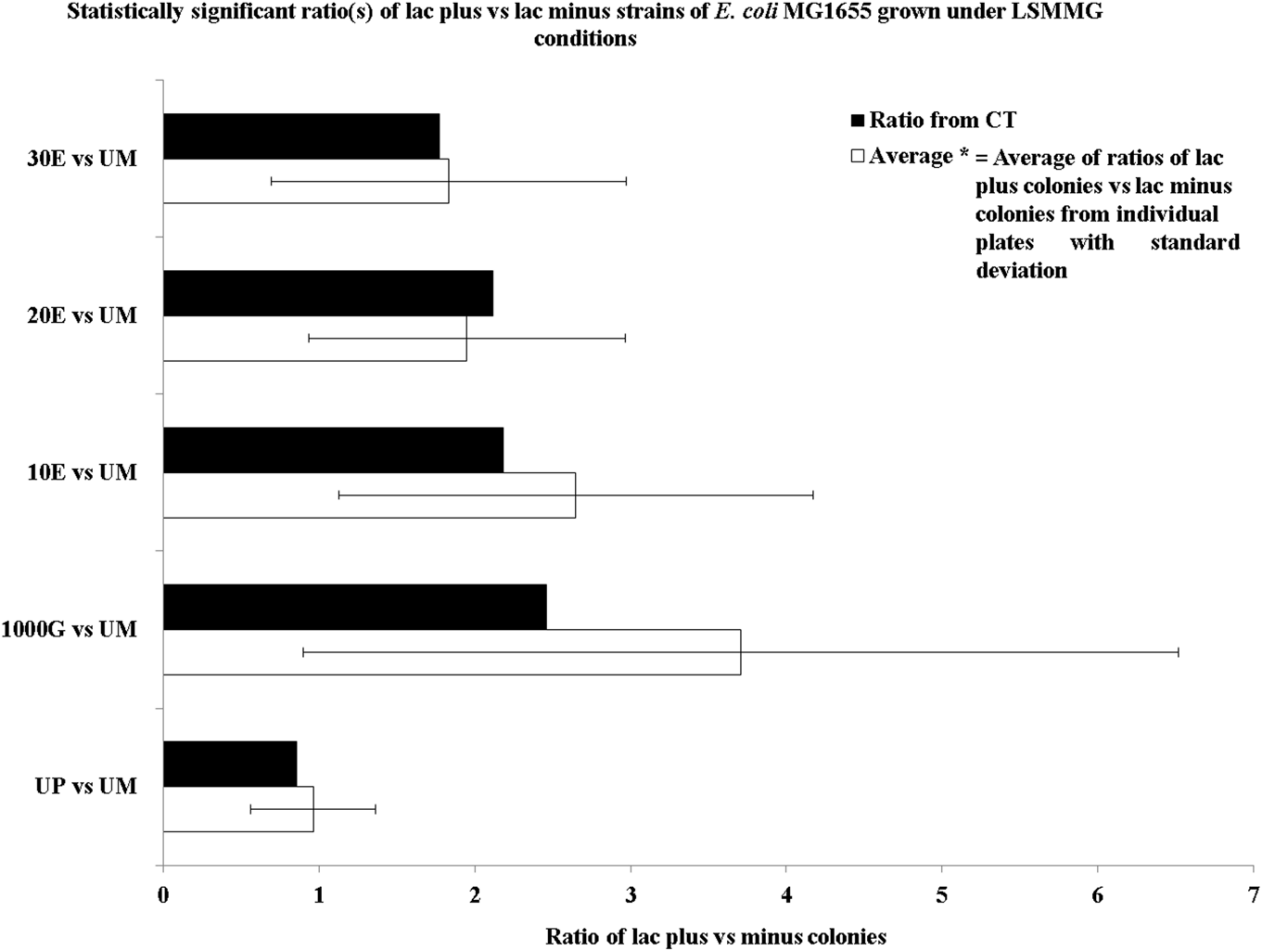
Ratio of cumulative totals (CT) of lac plus colonies vs. lac minus colonies from all the plates; Average * = Average of ratios of lac plus colonies vs. lac minus colonies from individual plates with SD; *G* = number of generations; 1000G = lac plus strain adapted to 1000 generations of LSMMG; 1000G-10E, 1000G-20E and 1000G-30E refer to the 1000G strain subjected to 10, 20 and 30 generations of ‘erasure’ on shaker flask conditions, respectively; *UP* unadapted lac plus, *UM* unadapted lac minus

To assess the extent of such genomic adaptation, isogenic strains of *E. coli* that can be readily distinguished by colony color were used in competition studies. Initially, the two strains were competed against one another by growing them together. After one cycle of growth, the mixture of cells was streaked onto multiple plates. Following incubation, the number of colonies of each type were counted on each plate to determine the ratio of the lac plus and lac minus strains. The ratios seen on individual plates varied widely with large standard deviations (Fig. 1, Supplementary Table 1). Thus, to calculate overall ratios, the colony counts were pooled from all the plates used in multiple trials/sets. Each set (trial) refers to a different experiment. For example, the nine ‘control’ sets, correspond to nine different flasks containing the combined growth of the lac plus and lac minus cultures. Under LSMMG/HARV conditions, the unadapted *E. coli* MG1655 lac plus strain and the unadapted *E. coli* MG1655 lac minus strain both grew nearly equally well with a lac plus/lac minus ratio of 0.86. When the long-term-adapted 1000-G strain was competed vs. the unevolved *E. coli* MG1655 lac minus strain, the lac plus/lac minus ratio increased dramatically to 2.46. However, if the 1000G strain were first grown under shake flask conditions for 10, 20, or 30 generations, the ratio initially declined to 2.19 and subsequently was 2.12 and 1.78 (Fig. 1). Thus, 72% of the adaptive advantage survived the extended erasure (Supplementary Fig. 1). An alternative approach is to first determine ratios for each individual plate and then calculate averages. This allows statistical validation whereas the first method does not.

Using this method, the respective ratios were 0.96 (with a robust standard deviation (SD) of 0.4 a coefficient of variation (CV) of 42%, and a confidence interval (CI) of 0.12), 3.71 (SD = 2.81, CV = 76%, CI = 0.9), 2.65 (SD = 1.52, CV = 57%, CI = 0.63), 1.95 (SD = 1.01, CV = 51%, CI = 0.46) and 1.83 (SD = 1.14, CV = 62%, CI = 0.49). The corresponding sample CV corrected for bias are 5.2, 8, 4.4, 2.9, and 3.9, respectively (Supplementary Table 1). Scatter plots performed on each of the datasets showed robust trend lines (Supplementary Fig. 2). In the overall analysis, approximately 50% of the advantage persists.

To further determine the variance differences, we applied the *F*-test to the lac plus vs lac minus ratio for two data sets. These were the unadapted lac plus strain vs. the unadapted lac minus strain and the 1000G-adapted lac plus strain vs. the unadapted lac minus strain. Given the vast variance differences between the two data sets as observed in the *F*-statistic value of 0.02 compared to the *F* Critical one-tail value of 0.6, we performed the *t*-test assuming unequal variances for this same pair of data sets. The *t*-test results show that the two-tailed and one-tailed *p*-values (9.2 × 10^−15^ and 1.8 × 10^−14^, respectively) are well under the statistical threshold of 0.05, thus clearly indicating the dominance of the 1000G plus strain over the unadapted lac minus strain when grown under LSMMG.

The paired *t*-test was then performed on each of the three data sets from the 1000G strain grown under shake flask conditions for 10, 20, or 30 generations (competed with the unadapted lac minus strain) against data from the 1000G-adapted strain grown with the unadapted lac minus strain. The results in all three cases show consistent two-tailed and one-tailed *p*-values of 0.5 and 1, respectively, thus, supporting our inference that the adaptation is only partially lost. A linear fit for the ratios (data sets) of 1000G strain grown under shake flask conditions for 10, 20, or 30 generations (competed with the unadapted lac minus strain) showed a trend consistent with our observations (Supplementary Fig. 3a). Similarly, a scatter plot of the same data set with the unadapted lac plus and minus ratio strain showed a correlation consistent with our observations (Supplementary Fig. 3b). The statistical values are provided in Supplementary Table 2.

In summary, extended exposure to the HARV conditions gave the MG1655 lac plus strain a significant adaptive advantage when the two strains were competed in the HARV. When the adapted strain was first exposed to shake flask conditions, the advantage was partially lost. However, even after three cycles of erasures, over 50% of the adaptation remained.

### Genome sequencing

**Fig. 2:**
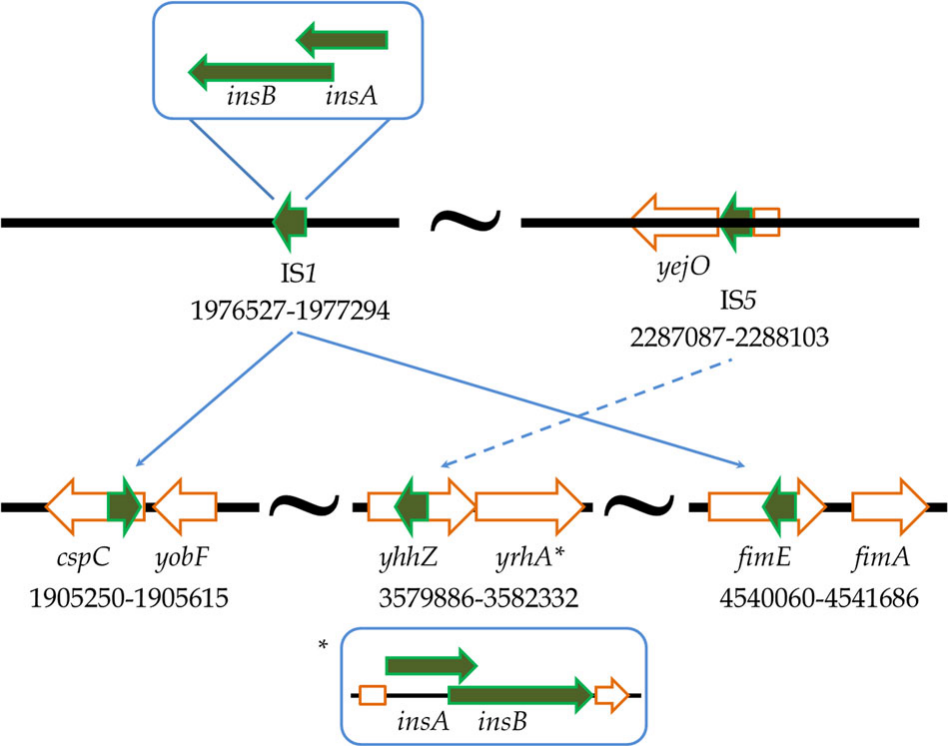
An insertion of the transposon IS1 to the left of position 1,905,401 in the forward orientation (+) is predicted (coordinate as annotated in NC_000913.2). At the point of insertion, it duplicates nine base pairs (1905401-1905409). Thus, the sequence goes up to 1,905,409 on the left side and then goes into the IS1 element, which is inverted, and then comes out at 1,905,401 and continues to higher coordinates. All of this occurs within the “*cspC*” gene, thereby likely making it dysfunctional. Likewise, transposon insertions have been predicted for positions 3,580,708 (four base pairs) and 4,540,601 (nine base pairs), disrupting the genes *yhhZ* (conserved protein) and *fimE* (tyrosine recombinase/inversion of on/off regulator of *fimA*), respectively. The JC (New junction) evidence thus shows the two new junctions (one on each side) that the mutations were predicted from. Two other transposon insertions occur in intergenic regions and may not have any impact on the actual genome itself. This figure was prepared by Quyen Tran

The genomic data gave no indication of contamination and the 16 S rRNA sequences were as expected for *E. coli*. Overall, 16 changes were found in the genome of the evolved 1000G *E. coli* MG1655 strain, compared to the control strain (Table 1). This included eight point mutations (six in genes *surA, betA, ygfK, trkH, fimH, ylbE*, and two intergenic (*cspH-cspG, and ppiC-yifN*), two deletions (one in the gene *fhuA* and the other intergenic *insB*-*insA*), and six insertions. Five of the insertions are mediated by transposons (*cspC, insA-uspC, gatY-fbaB, yhhZ, fimE*), while one is in the gene *ylbE*. There were five point mutations, resulting in amino acid changes, none of which were neutral. One large deletion eliminates 776 bases from an intergenic region (*insB*). A second one base deletion disrupts the reading frame of *fhu*A. In another case, a single base addition in combination with a single base point mutation converts a pseudogene (*ylbE*) into a potentially viable coding region. Five major transposon insertions occur at various loci, two of which are in intergenic non-coding regions, while three occur within coding genes. Of the three intragenic insertions, one (IS1) occurs within two genes involved in stress response and motility (Table 1). The description of the insertions as predicted by *breseq* is given in more detail in Fig. 2.

**Table 1:** Mutations found in *E. coli* MG1655 (lac plus) after 1000 generations of LSMMG

Position	Type of change	Mutation type	Annotation	Gene	Usual product	Function
54,033	G→T	Base change	Q224K (CAG→AAG)	*surA* ^a^ ←	Periplasmic peptidyl-prolyl isomerase	Outer membrane protein folding
326,446	C→T	Base change	G9D (GGT→GAT)	*betA* ←	Choline dehydrogenase (flavoprotein)	Protection against osmotic stress
3,015,315	G→A	Base change	G412R (GGA→AGA)	*ygfK* →	Possible oxidoreductase, Fe—S subunit	Putative selenate reductase
4,031,240	G→A	Base change	G25R (GGG→AGG)	*trkH* →	Potassium transporter	Transport
4,546,957	G→C	Base change	V43L (GTA→CTA)	*fimH* →	Minor type 1 fimbriae subunit	d-Mannose-specific adhesin
547,694	A→G	Base change	Pseudogene (139/252 nt)	*ylbE* ^c^ ←	C‑terminal fragment (pseudogene)	Two mutations make ORF
547,831	+G	Base insertion/addition	Pseudogene (2/252 nt)	*ylbE* ^c^ ←	C‑terminal fragment (pseudogene)	unknown function
169,483	Δ1 bp	Base deletion	Coding (2000/2244 nt)	*fhuA* →	ferrichrome outer membrane transporter	Outer membrane transport
1,976,527	Δ776 bp	Base deletion	Intergenic	*insB*–*insA*	–	–
1,050,465	T→A	Base change	Intergenic (−67/−219)	*cspH*←/→*cspG*	–	–
3,957,957	C→T	Base change	Intergenic (−121/+78)	*ppiC*←/←*yifN*	–	–
1,905,401	IS1 (+)+9 bp	Transposon MR^b^	coding (51–59/210 nt)	*cspC* ←	Deletes CspA-family stress protein	Stress
1,977,510	IS5 (+)+4 bp	Transposon MR	intergenic (−271/−264)	*insA*←/→*uspC*		–
2,175,275	IS1 (+)+9 bp	Transposon MR	intergenic (−49/+251)	*gatY*←/←*fbaB*		–
3,580,708	IS5 (–)+4 bp	Transposon MR	coding (823–826/1179 nt)	*yhhZ* →	Deletes conserved hypothetical protein	
4,540,601	IS1 (+)+9 bp	Transposon MR	coding (542–550/597 nt)	*fimE* →	Deletes tyrosine recombinase	Inversion of on/off fimA regulator

*IS* insertion sequence

^a^ functional gene product aids proper folding of outer membrane proteins OmpA, OmpF and LamB, disrupted and likely dysfunctional

^b^ Transposon-mediated rearrangement

^c^ function of product unknown

←gene orientation on reverse strand

→gene orientation on positive strand

←/→intergenic

Δ deletion

### Antibiotic susceptibility—VITEK results

No significant changes in antibiotic resistance were observed when comparing the unevolved *E. coli* lac plus strain to the same strain grown under LSMMG conditions for 100, 200, 300, 400, 500, 600, 700, 800, 900, and 1000 generations (Supplementary Table 3). The initial strain and all the evolved strains were sensitive to all the antibiotics used in the VITEK analysis. The antibiotics used in this study included ampicillin, amoxicillin/clavulanic acid, piperacillin/tazobactam, cefalotin, cefazolin, cefuroxime, cefuroxime axetil, cefoxitin, cefpodoxime, ceftazidime, ceftriaxone, cefepime, gentamicin, tobramycin, ciprofloxacin, levofloxacin, norfloxacin, tetracycline, nitrofurantoin, and trimethoprim/sulfamethoxazole. Thus, no increase in resistance was observed in the evolved as compared to the unevolved control strain (Supplementary Fig. 4, Supplementary Table 3).

## Discussion

The unevolved *E. coli* MG1655 lac plus strain does not initially possess a natural competitive (growth) advantage over the lac minus strain under LSMMG conditions. However, exposure to LSMMG over 1000 generations increases the fitness level of the lac plus strain. This is evident from the competition experiments in which the lac plus strain significantly outcompetes the unadapted *E. coli* MG1655 lac minus strain when both are grown together under LSMMG conditions. A portion of this adaptation is transient, which is clearly seen when the ability of the 1000G lac plus strain to outcompete the unadapted lac minus strain is decreased following erasure by overnight growth in the different environment found in shake flasks. However, repeated cycles of erasure have minimal additional effect, implying that there is a residual genomic component to the adaptation. Genomic adaptation to any new environment is in fact expected. In previous long term studies of *E. coli*, it was observed that the initial mutations were frequently adaptive rather than neutral.^22, 23^

Consistent with this, in the present case, genome re-sequencing revealed five changes in coding regions, none of which were neutral. In the case of *surA* (Q224K), *ygfK* (G412R), and *trkH* (G25R) a non-charged amino acid is converted to a basic residue. The gene *betA* undergoes a mutation changing a non-charged residue to an acidic residue (G9D). The fifth mutation in the gene *fimH* changes V43L with no net charge chang*e. surA, fimH*, and *betA* are of particular interest from an LSMMG adaptation perspective.

The protein encoded by *surA* plays a key role in pili biogenesis^39^ as well as the folding and assembly of fimbrial proteins^40–42^ on gram-negative bacterial surfaces, which enable attachment to surfaces of host cells and tissues for colonization and infection.^43, 44^ Thus *surA* indirectly influences bacterial adhesion. Furthermore, the SurA protein is conferred protection by the periplasmic acid stress chaperone HdeA,^45^ which was shown to be overexpressed in *E. coli* MG1655 when grown short-term under LSMMG.^34^ Thus, the *surA* mutation in the 1000G strain may have a key role in the response to LSMMG.

The gene *fimH* encodes a bacterial adhesion protein. FimH (along with other receptor/adhesion proteins) has been well-characterized for key structural and functional properties, as adaptation responses to increased shear stress on surfaces subject to differential flow dynamics.^46–49^ The V43L mutation is in the N-terminal lectin domain of FimH (N-FimH). Two residues, N46 and D47, in the vicinity of V43L, are important for the N-FimH domain’s binding and polymerization functions.^50^ Thus, it is possible that the V43L change in the N-FimH gene may influence its function.

The third gene, *betA*, is involved in osmotic stress response.^51, 52^ Surface-exposed fimbrial subunits are classified under the LSMMG regulon in *S. enterica* serovar Typhimurium, with direct implications for host immune system-pathogen interactions.^7^ The five observed non-synonymous mutations may be part of a regulon that facilitates adaptation of *E. coli* MG1655 strain to the LSMMG environment. Such adaptation may influence the bacterium’s ability to colonize the interior surfaces of the space station or spacecraft, including life support systems.

In addition, five changes are associated with insertion sequence (IS) elements. Such elements are known to increase the fitness of *E. coli* strains by mediating major transpositions in a genome, generating extensive genome rearrangements, and thereby increasing mutation rates and thus facilitating genome evolution.^53, 54^ The two insertions observed, IS1 and IS5, have both been well-studied in natural isolates of *E. coli* (the ECOR collection) with respect to their distribution and abundance.^55^ IS1 and IS5 are reported to be poorly regulated transposons and an increase in their copy number is correlated with a moderate to strong decrease in fitness.^56^ On the other hand, the IS1 element is also reported to increase mutational supply^57^ and occasionally generate variants with especially large phenotypic effects, such as host SOS response.^58^

Overall, the changes appear to be associated primarily with interaction with the environment. In other words, the cells are likely learning how to best interact with the low shear environment presented by the HARV. Thus, we see changes that are associated with transport, outer membrane interactions, and stress response. However, a 1000G non-LSMMG control is not available. Thus, the unadapted lac plus strain was used as the control for comparison of the sequencing results. Future studies are therefore needed to assess whether the mutations observed were specifically induced by exposure to the modeled microgravity environment.

Although adaptation to the LSMMG environment likely occurred, this did not impact antibiotic sensitivity. Previous experiments examined bacterial drug resistance in space flight samples. In these studies, a significant increase in the minimum inhibitory concentration levels of a few antibiotics was seen when tested against *E. coli* isolated from cosmonauts.^59–61^ However, bacteria, grown in culture for 4 months on Space Station Mir typically became less resistant to antibiotics.^14^ The present results suggest that antibiotic resistance strains likely did not develop as the result of adaptation to the low shear microgravity environment. Instead, the observed resistance to antibiotics seen in some earlier studies may reflect prior exposure of the natural microflora of the space station or the cosmonauts themselves.

Complex dynamics are often involved in long-term evolution of genomes.^62, 63^ Thus, 1000 generations is exceedingly unlikely to represent the end of adaptation. It can be expected that organisms on the space station or other long term missions would continue adapting indefinitely as has been observed in long-term studies of *E. coli*.^62, 64–69^ This process would possibly be accelerated by the elevated radiation levels experienced in space. During such true long-term adaptation, neutral mutations would likely become more common. At the very least, the initial focus of the adaptation would likely continue to be on the direct interaction with the low shear/microgravity environment and hence the types of changes seen here, rather than unrelated changes. However, the observation that there is enduring adaptation to the LSMMG environment supports the concern that organisms exposed to microgravity may as a by-product evolve undesirable properties such as predisposition to a growth stage that facilitates avoidance of the immune system.^17^

Monitoring microbial response to real time space conditions over extended periods is constrained by operational difficulty as well as costs, and thus, simulated microgravity offers a cheaper prototype analog of space conditions. Whether the results seen here reflect peculiarities of the HARV as a model system or an actual adaptation to microgravity is uncertain and would be best resolved by extended studies on the International Space Station. It is, however, reassuring that despite ongoing genomic adaptation to the LSMMG environment, increased antibiotic resistance was not observed.

## Methods

**Fig. 3:**
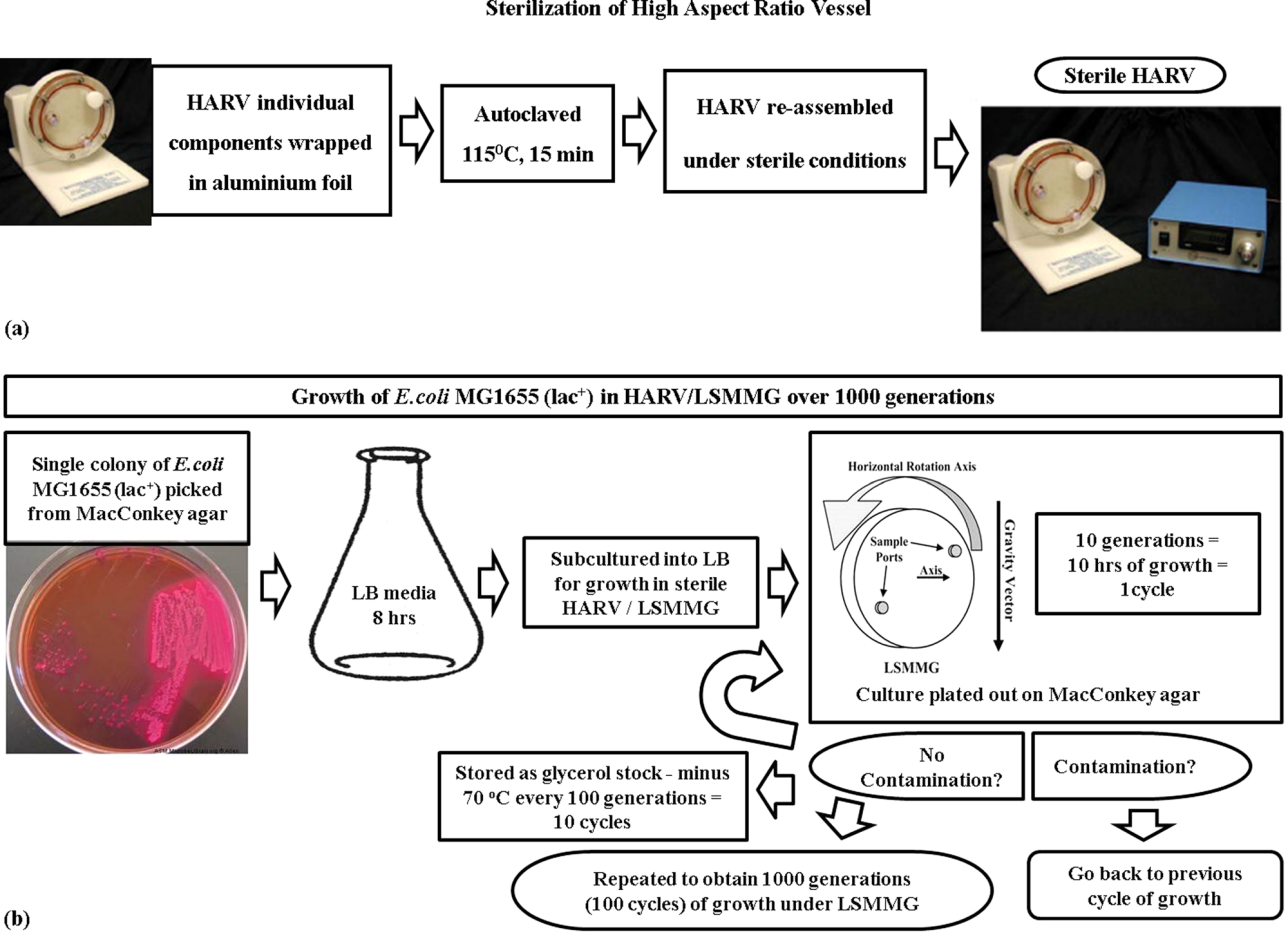
Overview of growth procedures: (**a**) Sterilization protocol for the HARV(s); (**b**) Protocol for growth of *E. coli* MG1655 lac plus strain in the HARV(s) This figure was prepared by Madhan Tirumalai

**Fig. 4:**
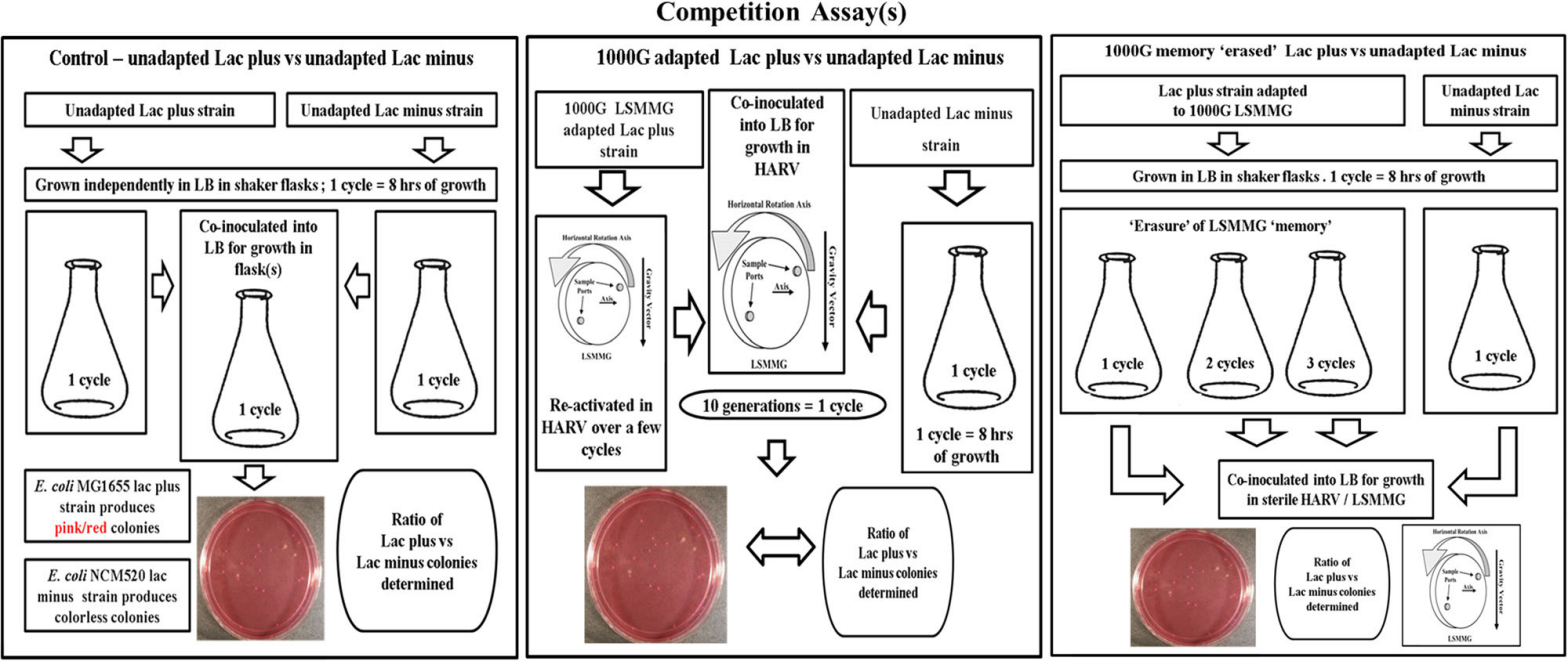
Competition assay(s) of *E. coli* MG1655 lac plus vs. lac minus under different conditions. This figure was prepared by Madhan Tirumalai

An overview of the experimental procedures is provided as Figs. 3 and 4.

### Cell culture

An isogenic pair of *E. coli* strains, namely NCM520 (designated genotype F^−^, *Δ*(*lacA-lacZ*)*880*(*FRT*), *λ* ^*−*^, *rph*-1; CGSC # 8252) a lac minus strain derived from MG1655 in which the entire lac operon was deleted and the well characterized MG1655 (a lac plus strain; CGSC # 6300)^70^ were obtained from the *E. coli* Genetic Stock Center at Yale University.^71^ The MG1655 strain was selected for analysis because its genome has been completely sequenced and is well annotated.^72^ The two strains can be easily differentiated on MacConkey agar medium by colony color.^73^ Both strains were immediately and separately inoculated into Luria Broth (LB) medium^74^ and incubated at 37 °C, 150 rpm on a rotary shaker. All shaker culture growth studies were conducted at these standard conditions. At saturation, the cultures were maintained as glycerol stocks at −70 °C for later use.

### Simulated microgravity/LSMMG

In order to obtain simulated microgravity, cells were grown in HARVs (HARVs) (Synthecon Inc., Houston, TX) at 37 °C in 50 ml of LB medium. The LSMMG environment was obtained by HARV rotation at 25 rpm on a horizontal axis.^6, 7^ The aerobic environment is maintained by gas exchange which occurs by perfusion through a permeable membrane. Cell growth was monitored spectrophotometrically (DU530 spectrophotometer; Beckman Instruments, Fullerton, CA) at 600 nm by removing 500-µl samples every 20–45 min without interrupting culture agitation; no more than 5% of the culture was removed prior to harvesting of cells for DNA isolation.

### Preparation of HARV vessels

The HARV-vessels were thoroughly rinsed first under running water. The entire set of different parts of the HARV unit was placed in a tub, and were treated with 25% bleach solution and left standing for 10–15 min. The bleach-treated parts were then rinsed thoroughly under running water and loosely reassembled, wrapped in aluminum foil and autoclaved at 117 °C for 15 min. The sterile HARVs were allowed to cool under sterile conditions, the vessels completely reassembled and then used for growth studies (Fig. 3a).

### Growth of the *E. coli* MG1655 lac plus strain over 1000 generations of LSMMG exposure

A single colony was picked from an overnight LB agar plate and inoculated into LB medium for growth in an autoclaved HARV. Initially, growth curves were obtained for the *E. coli* MG1655 lac plus strain in the HARV (Supplementary Fig. 5) and the number of generations were inferred to be approximately ten for each cycle, Each cycle of growth reached saturation over 8–12 h, and the cells were then removed and treated as follows. One aliquot of 500 µl was preserved as glycerol stock. A second aliquot of 10 µl was diluted appropriately and plated on LB agar plates for growth in an incubator at 37 °C to rule out contamination. If contamination was detected, the growth was reverted back to the previous stage/cycle to ensure contamination free adaptation. A third aliquot of 500 µl was used to subculture cells into LB medium for growth in a sterile HARV. This procedure was continued for approximately 100 growth cycles. Each cycle represented approximately ten generations of growth resulting in approximately 1000 generations of growth for each original culture (Fig. 3b). Plating out was done as follows: the cell cultures were serial diluted and 50 µl of the same added to the center of the plate. Sterile glass beads numbering 6–7 were added to the plate and the plate was placed on a flat surface and shaken to spread the 50 µl of the diluted culture.

### Competition growth studies of the isogenic pair

When grown on MacConkey agar, the MG1655 lac plus strain produces pink colonies and the NCM520 lac minus strain produces colorless colonies. A single colony of each strain was picked from MacConkey agar plates streaked with the strain(s) overnight from the glycerol stocks and inoculated separately in LB and set for growth. At growth saturation (O.D of 1.25 at A_600_), 5 μl each of the two strains were combined and inoculated into 50 ml of LB for growth in five sets of flasks. The growth of the strains was quantified by plating them out at saturation on MacConkey agar. The cultures were allowed to grow until they reached the end of the log phase, which based on growth studies (Supplementary Fig. 5) is equivalent to ten generations. The culture was only then plated out. The plates were placed in a 37 °C incubation chamber overnight. The ratio of the lac plus and lac minus colonies was determined by counting the numbers of colonies of each color on each plate. Because the number of colonies per plate is highly variable, we calculated the ratio based on the total counts from all the plates. Alternatively, the ratio for each plate was determined and these could then be averaged.

### Competition growth studies of the isogenic pair under microgravity conditions

The lac plus strain evolved over 1000 generations (1000G) under microgravity (LSMMG) in HARV were directly inoculated into LB medium and set for growth under appropriate LSMMG conditions for several cycles to reactivate growth. The reactivated lac plus strain was co-inoculated with an equal amount of the lac minus strain grown in LB medium in a flask at 37 °C overnight. Care was taken to ensure both cultures had grown to similar OD levels, in order to obtain equal amounts of cells. LB medium was inoculated with this mixed population grown under LSMMG conditions in the HARV. The ratio of the lac plus strain (pink colonies) to the lac minus strain (white colonies) was again determined.

### Competition growth studies of the unevolved isogenic pair under microgravity conditions

The above was repeated, but instead of the 1000G lac plus strain, the unevolved *E. coli* MG1655 lac plus (WT) strain grown in LB under shaker flask conditions was co-inoculated with an equal amount of the lac minus strain that was grown in LB medium in a flask at 37 °C overnight.

### Adaptation ‘erasure’ experiment

The LSMMG 1000G-adapted strain and the lac minus strain were grown in LB medium in a flask under rotary conditions at 37 °C overnight. The two strains were co-inoculated and the competition assay was repeated under LSMMG at 37 °C, and the ratio of lac plus and lac minus was calculated (Fig. 4). This procedure was repeated after exposing the 1000G LSMMG adapted strain to shake flask conditions for two and three cycles (one cycle referring to ten generations of growth), respectively to determine if repeated exposure of the lac plus strain to shake flask conditions would erase the adaptation.

### VITEK analysis for measuring antibiotic sensitivity

The antibiotic sensitivity of the *E. coli* strain(s) exposed to different conditions were assayed using the VITEK^®^ 2 Compact instruments and VITEK^®^ 2 PC software (BioMérieux, Inc., Hazelwood, MO). The antibiotics used in the VITEK analysis included ampicillin, amoxicillin/clavulanic acid, piperacillin/tazobactam, cefalotin, cefazolin, cefuroxime, cefuroxime axetil, cefoxitin, cefpodoxime, ceftazidime, ceftriaxone, cefepime, gentamicin, tobramycin, ciprofloxacin, levofloxacin, norfloxacin, tetracycline, nitrofurantoin, trimethoprim/sulfamethoxazole.

### Genome sequencing

The genome of the 1000G strain was examined for mutations using the Illumina Genome Analyzer System (Illumina^®^, Hayward, CA). Briefly, the genomic DNA sample preparation kit was used to build DNA libraries for single-read sequencing. Random size-selected fragments of DNA were created, polished, and prepared for the addition of unique adapters to each end of the DNA fragments. Standard Cluster Generation Kit reagents were used to bind DNA library samples to complementary adapter oligomers that were next grafted on the flow cell surface. This allowed amplification of the attached DNA fragments to create clonal clusters of roughly 1000 copies each. An Illumina Sequencing Kit was used to determine the DNA sequence of Clusters on the flow cell. All the sequencing steps were performed following the manufacturer’s specifications (Illumina^®^, Hayward, CA).

### Analysis of genome sequencing data

The individual reads were assembled de novo using Velvet.^75^ They were next aligned against the wild type *E. coli* MG1655 (CGSC7740) genome sequence using Mosaik^76^ and breseq.^77^ Mutation discovery was performed with MUMmer’s nucmer tool^78^ and gigaBayes.^79^ Each candidate mutation was visually checked and confirmed using Samtools’ tview^80^ to visualize the Mosaik alignment. As a result, a list of mutations within the 1000G HARV genome was created and further analyzed in order to evaluate the likely impact of the mutation at the protein level. Mutations found to be located within a gene were translated to assess any change at the amino acid level as compared to the original protein sequence using BioEdit (Hall, Ibis Biosciences, Carlsbad, CA). Online protein domain annotation tools such as SMART (version 7) or InterProScan were used to predict the location of the mutated amino acid position in the protein’s conserved domain(s)/motif(s).

## Electronic supplementary material

Supplementary Table 1

Supplementary Table 2

Supplementary Table 3

Figure S1

Figure S2

Figure S3a

Figure S3b

Figure S4

Figure S5
