# Supplementary material for: The adaptation of Escherichia coli cells grown in simulated microgravity for an extended period is both phenotypic and genomic
Source: NPJ Microgravity. 2017 May 23;3:15. doi: 10.1038/s41526-017-0020-1 (PMC5460176; doi:10.1038/s41526-017-0020-1)
Supplement: Supplementary file 1 — Supplementary Table 1 [file 41526_2017_20_MOESM1_ESM.pdf]

Supplementary Table 1: Colony counts and ratio(s) of lac plus vs minus

Media - LB
Cultures - 1000G Lac plus (reactivated over three cycles of growth in HARV) and lac minus together
Growth condition/environment - LSMMG (HARV)
CV - Coefficient of variation
CVB - CV corrected for bias
CT - Cumulative total colony counts pooled from all the plates used in multiple trials/sets
CI - Confidence Interval

	Control		
Set	Plus	Minus	Ratio
1	22	29	0.76
	22	28	0.79
	56	53	1.06
	33	27	1.22
	90	78	1.15
2	58	62	0.94
	27	26	1.04
	7	15	0.47
	17	32	0.53
	43	72	0.60
3	27	34	0.79
	33	32	1.03
	22	17	1.29
	48	36	1.33
	60	49	1.22
4	23	15	1.53
	14	22	0.64
	15	13	1.15
	52	34	1.53
	80	53	1.51
5	56	36	1.56
	39	25	1.56
	46	19	2.42
	49	52	0.94
	66	45	1.47
6	86	82	1.05
	60	82	0.73
	48	44	1.09
	76	80	0.95
	67	74	0.91
7	28	67	0.42
	69	70	0.99
	68	92	0.74
	31	33	0.94
	97	113	0.86
8	101	105	0.96
	30	49	0.61
	74	103	0.72
	87	120	0.73
	90	110	0.82
9	101	107	0.94
	62	89	0.70
	52	116	0.45
	53	115	0.46
	64	140	0.46
	23	78	0.29
		Average	0.96
		Std Dev	0.40
		CV	42%
		CVB	5.2
CT	2372	2773	0.86
		CI	0.12

	No Erasure		
Set	plus	minus	Ratio
1	45	13	3.46
	45	26	1.73
	64	49	1.31
	59	51	1.16
2	35	15	2.33
	62	29	2.14
	58	43	1.35
	66	50	1.32
3	58	49	1.18
	50	17	2.94
	66	21	3.14
	39	13	3.00
4	116	50	2.32
	118	43	2.74
	49	5	9.80
	78	13	6.00
5	60	6	10.00
	71	11	6.45
	78	10	7.80
	14	5	2.80
6	104	11	9.45
	21	3	7.00
	148	15	9.87
	14	2	7.00
7	25	4	6.25
	3	1	3.00
	13	3	4.33
	4	1	4.00
8	70	36	1.94
	79	45	1.76
	51	23	2.22
	52	32	1.63
9	55	32	1.72
	46	21	2.19
	40	25	1.60
	75	39	1.92
	7	11	0.64
	28	19	1.47
		Average	3.71
		Std Dev	2.81
		CV	76%
		CVB	8
CT	2066	842	2.46
		CI	0.9

10 generation Erasure

Set	plus	minus	Ratio
1	54	12	4.50
	43	11	3.91
	49	19	2.58
2	21	4	5.25
	56	15	3.73
	44	11	4.00
3	26	7	3.71
	52	11	4.73
	64	28	2.29
4	69	27	2.56
	39	29	1.34
	26	29	0.90
5	34	42	0.81
	31	57	0.54
	33	39	0.85
6	21	19	1.11
	11	27	0.41
	16	28	0.57
7	81	47	1.72
	29	37	0.78
	121	34	3.56
8	148	34	4.35
	84	24	3.50
	94	24	3.92
9	132	41	3.22
	102	26	3.92
	52	19	2.74
		Average	2.65
		Std Dev	1.52
		CV	57%
		CVB	4.4
CT	1532	701	2.19
		CI	0.63

20 generation Erasure

Set	plus	minus	Ratio
1	105	36	2.92
	89	28	3.18
	123	40	3.08
	99	37	2.68
2	116	40	2.90
	93	27	3.44
	81	43	1.88
	87	50	1.74
3	126	41	3.07
	80	34	2.35
	26	31	0.84
	22	21	1.05
4	11	19	0.58
	10	23	0.43
	9	10	0.90
	17	11	1.55
5	10	4	2.50
	29	25	1.16
	21	25	0.84
		Average	1.95
		Std Dev	1.01
		CV	51%
		CVB	2.9
CT	1154	545	2.12
		CI	0.46

30 generation Erasure

Set	plus	minus	Ratio
1	5	3	1.67
	2	7	0.29
	2	4	0.50
	3	4	0.75
2	3	2	1.50
	1	9	0.11
	4	3	1.33
	11	16	0.69
3	3	2	1.50
	48	37	1.30
	12	4	3.00
	57	37	1.54
4	11	4	2.75
	42	12	3.50
	34	14	2.43
	41	21	1.95
5	28	8	3.50
	39	9	4.33
	42	15	2.80
	37	25	1.48
	42	27	1.56
		Average	1.83
		Std Dev	1.14
		CV	62%
		CVB	3.9
CT	467	263	1.78
		CI	0.49
