# Supplementary material for: The adaptation of Escherichia coli cells grown in simulated microgravity for an extended period is both phenotypic and genomic
Source: NPJ Microgravity. 2017 May 23;3:15. doi: 10.1038/s41526-017-0020-1 (PMC5460176; doi:10.1038/s41526-017-0020-1)
Supplement: Supplementary file 2 — Supplementary Table 2 [file 41526_2017_20_MOESM2_ESM.pdf]

Supplementary Table 2: Statistics

t-Test: Two-Sample Assuming Unequal Variances

	Unadapted plus vs unadapted minus	1000G adapted plus vs unadapted minus
Mean	0.96383092	3.709766336
Variance	0.161629811	7.876749342
Observations	46	38
Hypothesized Mean Difference	2.75	
df	38	
t Stat	-11.97043276	
P(T<=t) one-tail	9.18612E-15	
t Critical one-tail	1.68595446	
P(T<=t) two-tail	1.83722E-14	
t Critical two-tail	2.024394164	

	1000G adapted plus vs unadapted minus	1000G plus 10G erasure vs unadapted minus
Mean	3.709766336	2.647935788
Variance	7.876749342	2.319080773
Observations	38	27
Hypothesized Mean Difference	1.06	
df	59	
t Stat	0.003380787	
P(T<=t) one-tail	0.498656966	
t Critical one-tail	1.671093032	
P(T<=t) two-tail	0.997313933	
t Critical two-tail	2.000995378	

	1000G adapted plus vs unadapted minus	1000G plus 20G erasure vs unadapted minus
Mean	3.709766336	1.951879174
Variance	7.876749342	1.027445008
Observations	38	19
Hypothesized Mean Difference	1.76	
df	52	
t Stat	-0.004132829	
P(T<=t) one-tail	0.498359152	
t Critical one-tail	1.674689154	
P(T<=t) two-tail	0.996718303	
t Critical two-tail	2.006646805	

	1000G adapted plus vs unadapted minus	1000G plus 30G erasure vs unadapted minus
Mean	3.709766336	1.832000215
Variance	7.876749342	1.300519825
Observations	38	21
Hypothesized Mean Difference	1.88	
df	54	
t Stat	-0.004305385	
P(T<=t) one-tail	0.498290338	
t Critical one-tail	1.673564906	
P(T<=t) two-tail	0.996580676	
t Critical two-tail	2.004879288	
