# Supplementary material for: The adaptation of Escherichia coli cells grown in simulated microgravity for an extended period is both phenotypic and genomic
Source: NPJ Microgravity. 2017 May 23;3:15. doi: 10.1038/s41526-017-0020-1 (PMC5460176; doi:10.1038/s41526-017-0020-1)
Supplement: Supplementary file 3 — Supplementary Table 3 [file 41526_2017_20_MOESM3_ESM.pdf]

Supplementary Table 3: VITEK results showing the antibiotic sensitivity of *E. coli* MG1655 lac plus strain evolved through 1000 generations in LSMMG

1C = 10 generations of growth **S = Sensitive** **MIC = Minimum Inhibitory Concentration**

	MIC (µg/ml)				
	**Ampicillin**	**Amoxicillin/Clavulanic Acid**	**Piperacillin/Tazobactam**	**Cefalotin**	**Cefazolin**
**Lac plus control**	S-4	S-8	S<=4	S=4	S<=4
**10C**	S<=2	S-4	S<=4	S-4	S<=4
**20C**	S<=2	S-4	S<=4	S-4	S<=4
**30C**	S-4	S-4	S<=4	S-8	S<=4
**40C**	S-8	S-4	S<=4	S-8	S<=4
**50C**	S-4	S-4	S<=4	S-8	S<=4
**60C**	S<=2	S-4	S<=4	S-4	S<=4
**70C**	S<=2	S-4	S<=4	S-4	S<=4
**80C**	S<=2	S-4	S<=4	S-4	S<=4
**90C**	S-8	S-4	S<=4	S-4	S<=4
**100C**	S-4	S-4	S<=4	S-4	S<=4

	**Cefuroxime**	**Cefuroxime Axetil**	**Cefoxitin**	**Cefpodoxime**	**Ceftazidime**
**Lac plus control**	S-4	S-4	S<=4	S<=0.25	S<=1
**10C**	S-4	S-4	S<=4	S<=0.25	S<=1
**20C**	S-4	S-4	S<=4	S<=0.25	S<=1
**30C**	S-4	S-4	S<=4	S<=0.5	S<=1
**40C**	S-4	S-4	S<=4	S<=0.5	S<=1
**50C**	S-4	S-4	S<=4	S<=0.25	S<=1
**60C**	S-4	S-4	S<=4	S<=0.25	S<=1
**70C**	S-4	S-4	S<=4	S<=0.25	S<=1
**80C**	S-4	S-4	S<=4	S<=0.25	S<=1
**90C**	S-4	S-4	S<=4	S<=0.25	S<=1
**100C**	S-4	S-4	S<=4	S<=0.25	S<=1

	Ceftriaxone	Cefepime	Gentamicin	Tobramycin	Ciprofloxacin
Lac plus control	S<=1	S<=1	S<=1	S<=1	S<=0.25
10C	S<=1	S<=1	S<=1	S<=1	S<=0.25
20C	S<=1	S<=1	S<=1	S<=1	S<=0.25
30C	S<=1	S<=1	S<=1	S<=1	S<=0.25
40C	S<=1	S<=1	S<=1	S<=1	S<=0.25
50C	S<=1	S<=1	S<=1	S<=1	S<=0.25
60C	S<=1	S<=1	S<=1	S<=1	S<=0.25
70C	S<=1	S<=1	S<=1	S<=1	S<=0.25
80C	S<=1	S<=1	S<=1	S<=1	S<=0.25
90C	S<=1	S<=1	S<=1	S<=1	S<=0.25
100C	S<=1	S<=1	S<=1	S<=1	S<=0.25

	Levofloxacin	Norfloxacin	Tetracycline	Nitrofurantoin	Trimethoprim/ Sulfamethoxazole
Lac plus control	S<=0.12	S<=0.5	S<=1	S<=16	S<=20
10C	S<=0.12	S<=0.5	S<=1	S<=16	S<=20
20C	S<=0.12	S<=0.5	S<=1	S<=16	S<=20
30C	S<=0.12	S<=0.5	S<=1	S<=16	S<=20
40C	S<=0.12	S<=0.5	S<=1	S<=16	S<=20
50C	S<=0.12	S<=0.5	S<=1	S<=16	S<=20
60C	S<=0.12	S<=0.5	S<=1	S<=16	S<=20
70C	S<=0.12	S<=0.5	S<=1	S<=16	S<=20
80C	S<=0.12	S<=0.5	S<=1	S<=16	S<=20
90C	S<=0.12	S<=0.5	S<=1	S<=16	S<=20
100C	S<=0.12	S<=0.5	S<=1	S<=16	S<=20
