# Supplementary figures and images for: The adaptation of Escherichia coli cells grown in simulated microgravity for an extended period is both phenotypic and genomic
Source: NPJ Microgravity. 2017 May 23;3:15. doi: 10.1038/s41526-017-0020-1 (PMC5460176; doi:10.1038/s41526-017-0020-1)

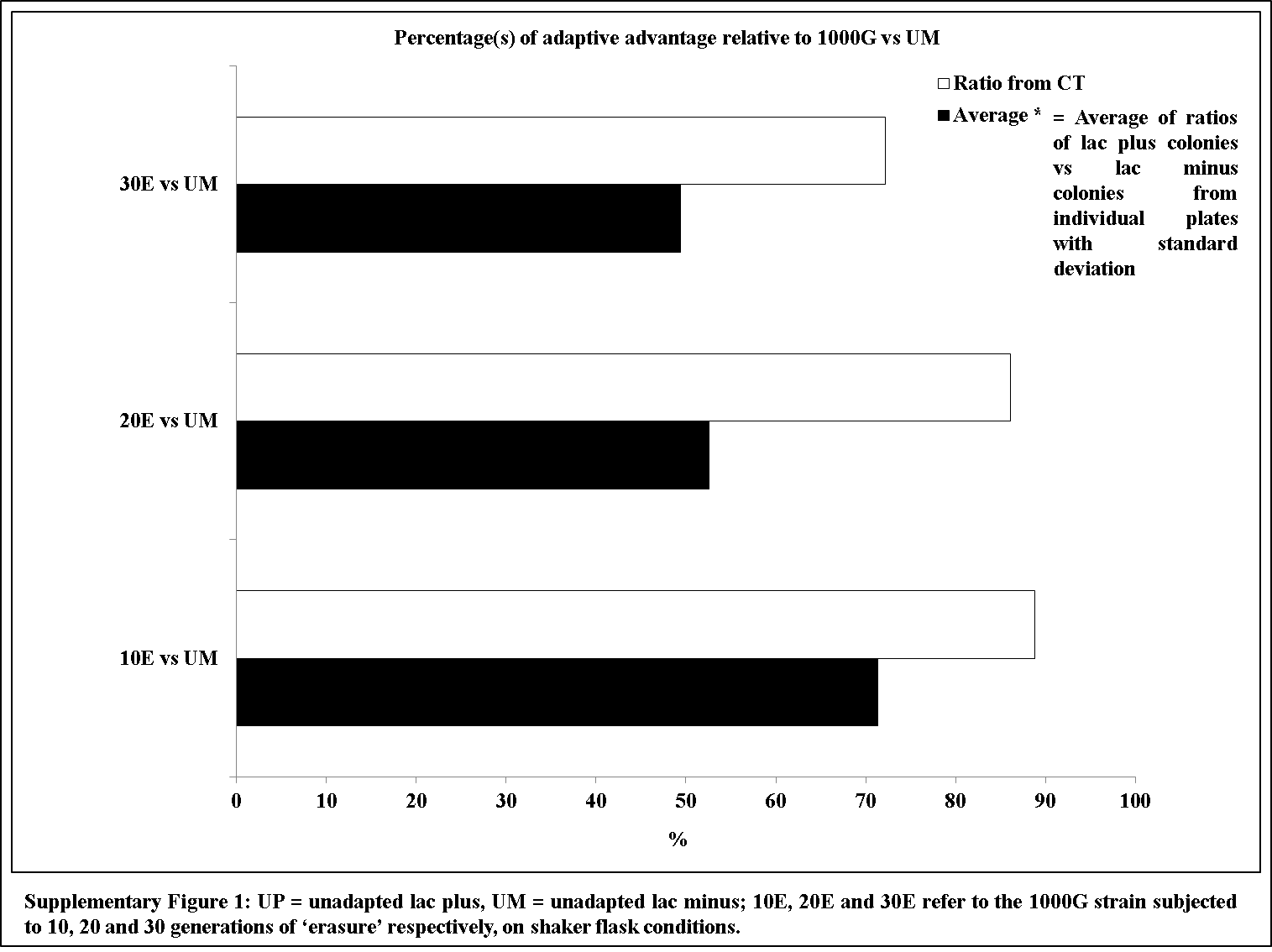

Supplement: Supplementary file 4 — Figure S1 [file 41526_2017_20_MOESM4_ESM.tif]

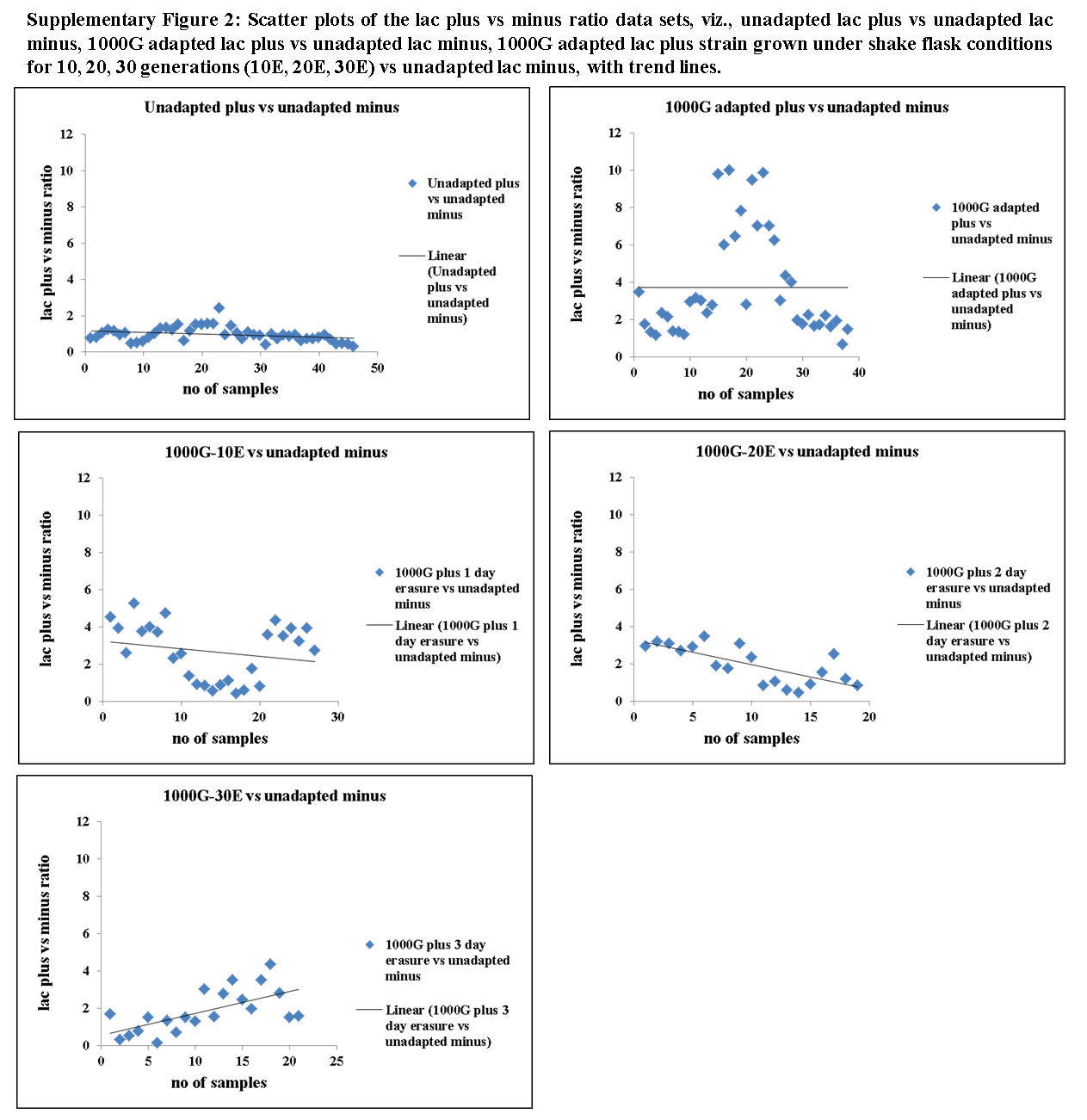

Supplement: Supplementary file 5 — Figure S2 [file 41526_2017_20_MOESM5_ESM.tif]

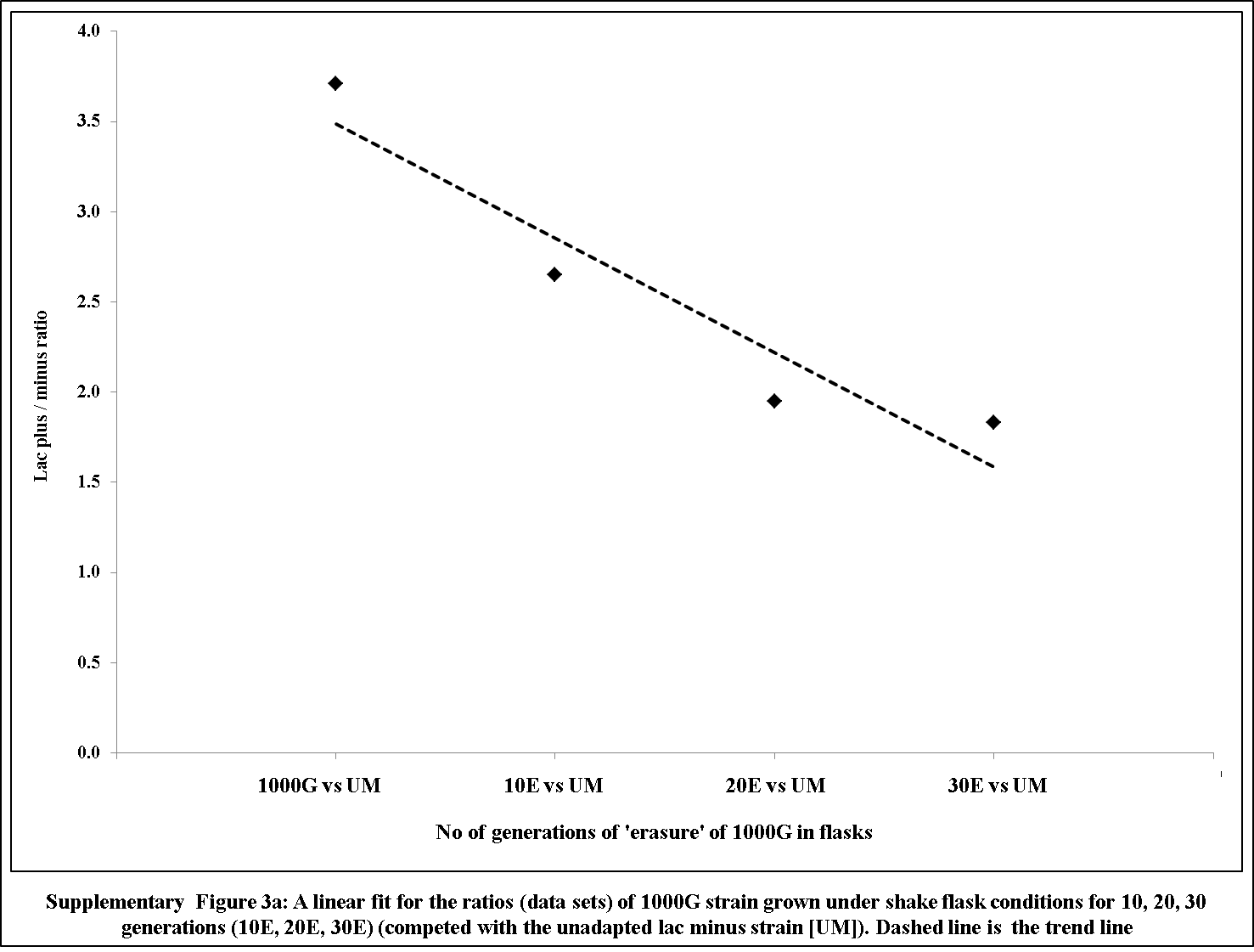

Supplement: Supplementary file 6 — Figure S3a [file 41526_2017_20_MOESM6_ESM.tif]

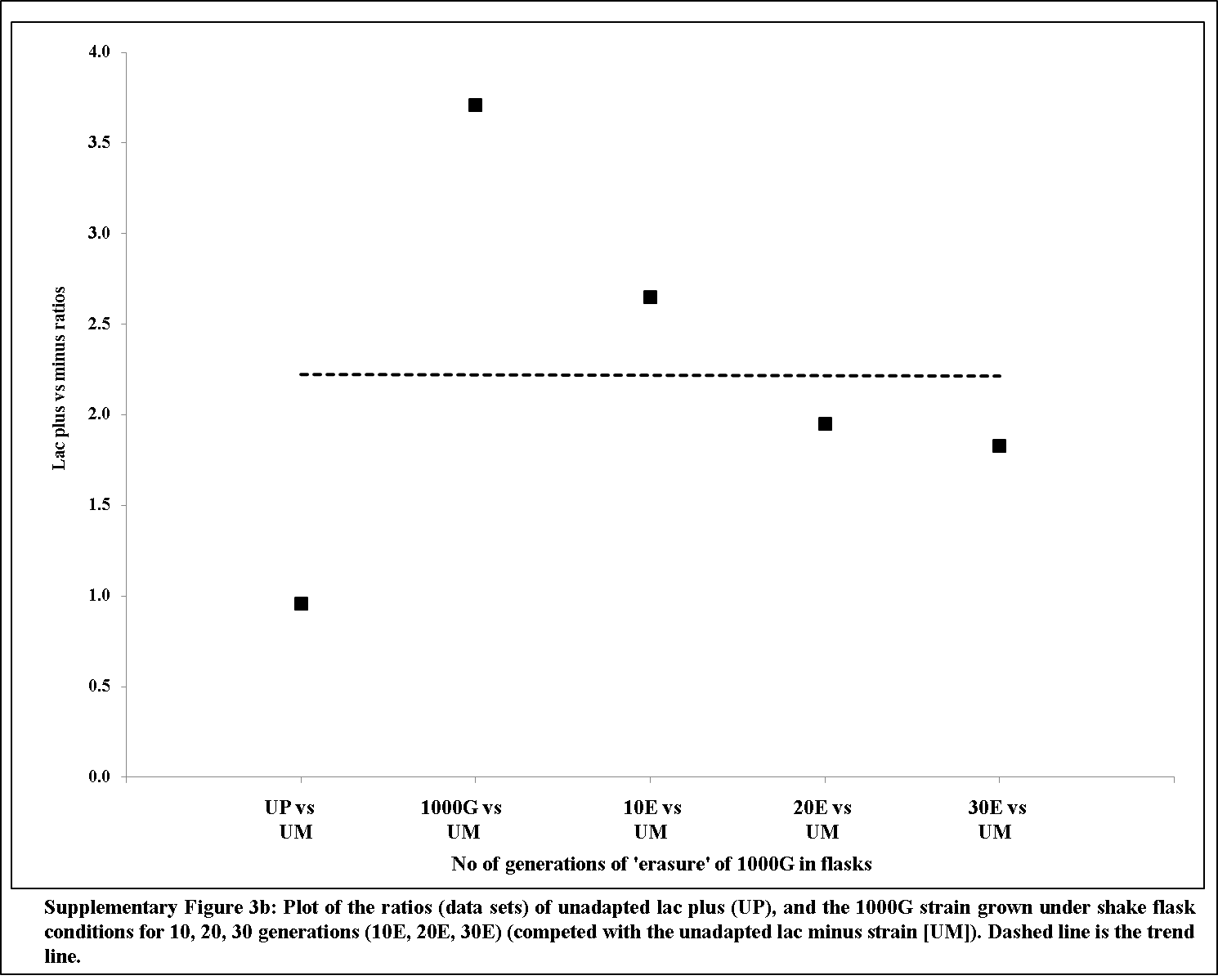

Supplement: Supplementary file 7 — Figure S3b [file 41526_2017_20_MOESM7_ESM.tif]

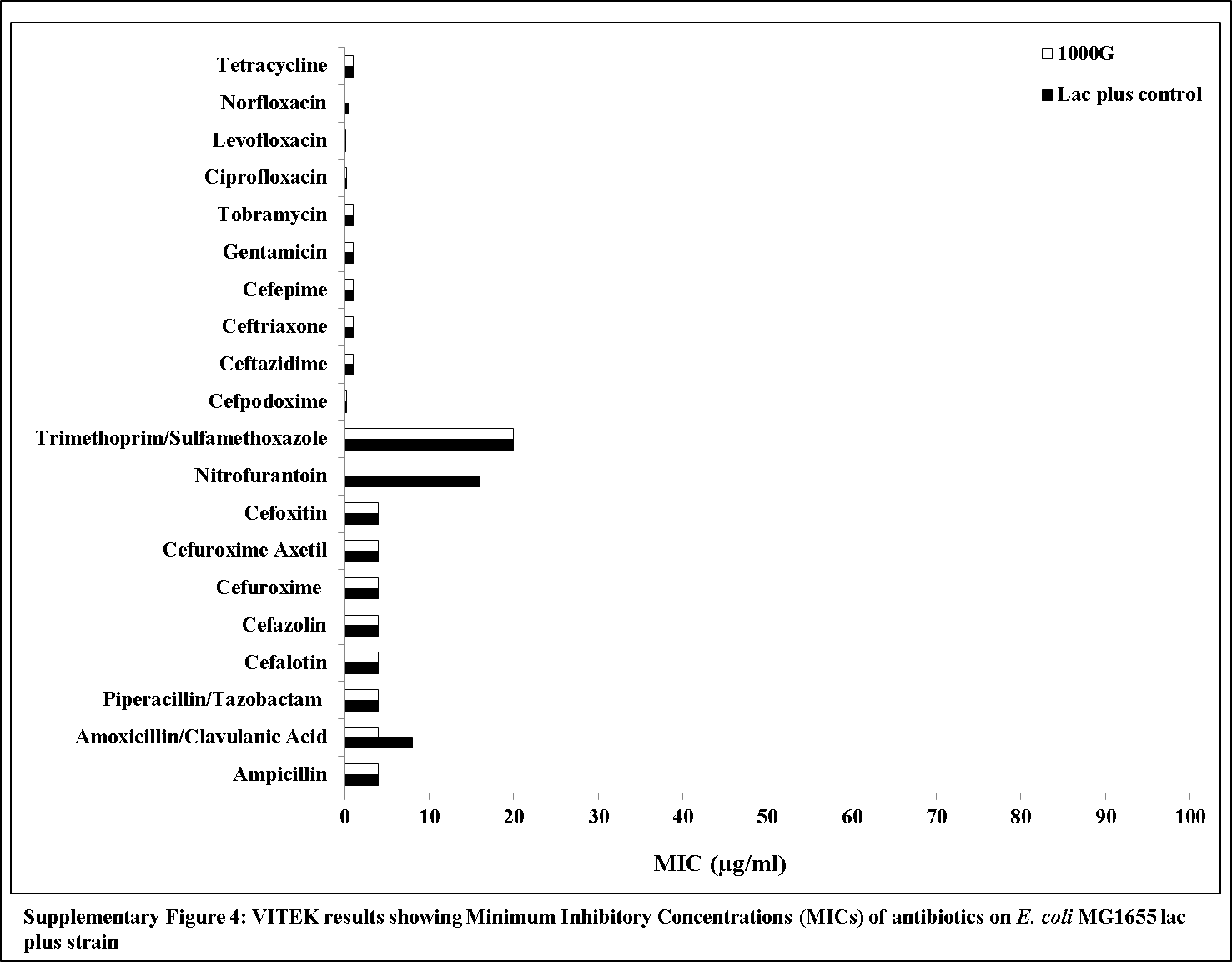

Supplement: Supplementary file 8 — Figure S4 [file 41526_2017_20_MOESM8_ESM.tif]

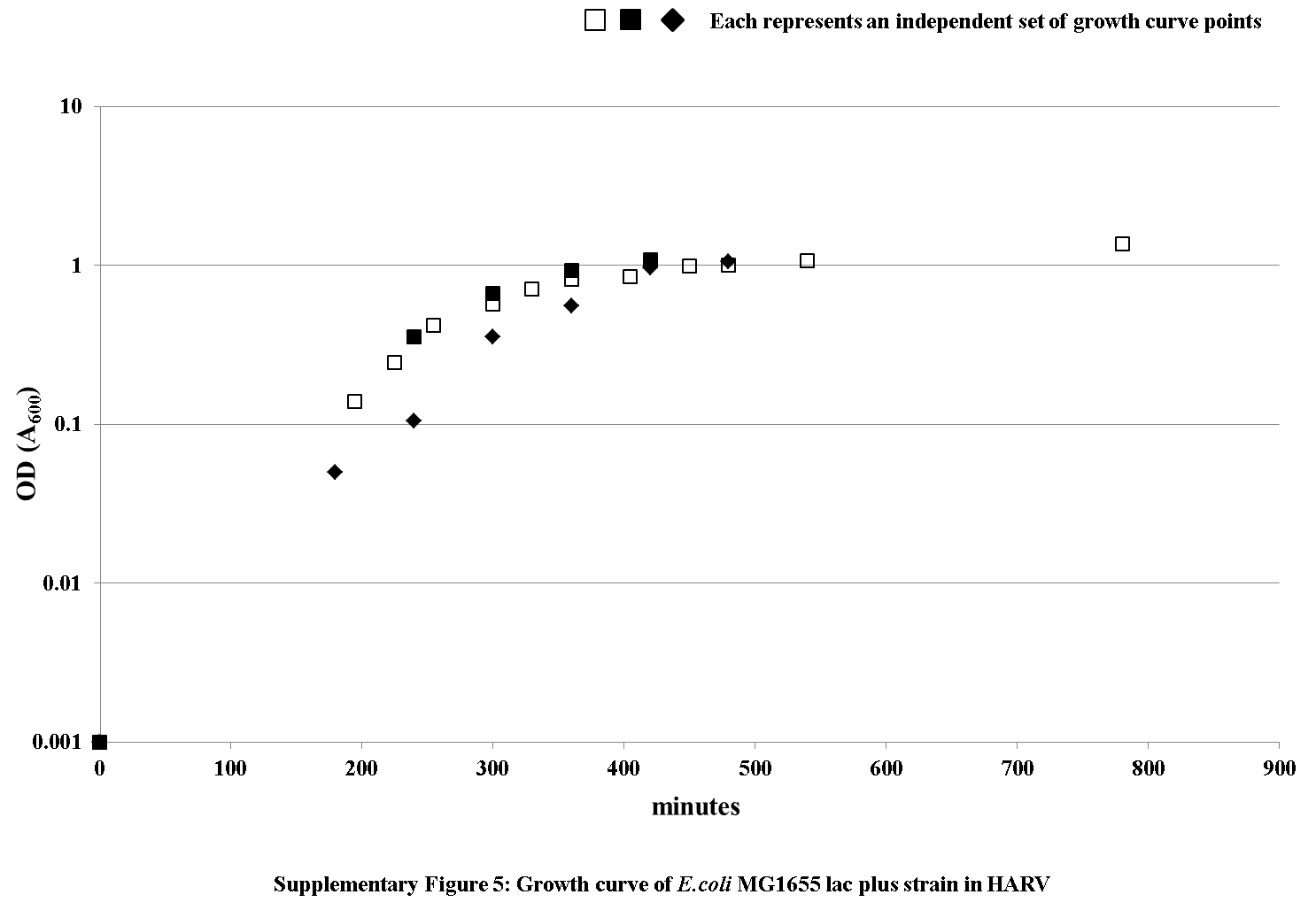

Supplement: Supplementary file 9 — Figure S5 [file 41526_2017_20_MOESM9_ESM.tif]
